# Lack of aggression and apparent altruism towards intruders in a primitive termite

**DOI:** 10.1098/rsos.160682

**Published:** 2016-11-09

**Authors:** Feargus Cooney, Emma I. K. Vitikainen, Harry H. Marshall, Wilmie van Rooyen, Robert L. Smith, Michael A. Cant, Nicole Goodey

**Affiliations:** 1 Centre for Ecology and Conservation, University of Exeter, Penryn Campus, Cornwall TR10 9EZ, UK; 2 Department of Entomology, University of Arizona, Forbes 410, Tucson, AZ 85721-0036, USA

**Keywords:** intergroup competition, cooperation, nest-mate recognition, allogrooming, aggression, *Pterotermes occidentis*

## Abstract

In eusocial insects, the ability to discriminate nest-mates from non-nest-mates is widespread and ensures that altruistic actions are directed towards kin and agonistic actions are directed towards non-relatives. Most tests of nest-mate recognition have focused on hymenopterans, and suggest that cooperation typically evolves in tandem with strong antagonism towards non-nest-mates. Here, we present evidence from a phylogenetically and behaviourally basal termite species that workers discriminate members of foreign colonies. However, contrary to our expectations, foreign intruders were the recipients of more rather than less cooperative behaviour and were not subjected to elevated aggression. We suggest that relationships between groups may be much more peaceable in basal termites compared with eusocial hymenoptera, owing to energetic and temporal constraints on colony growth, and the reduced incentive that totipotent workers (who may inherit breeding status) have to contribute to self-sacrificial intergroup conflict.

## 1. Introduction

One of the key mechanisms proposed to explain the evolution of altruism is the ability to direct care preferentially towards kin [1]. In primitively eusocial insects, colonies are typically composed of close relatives, in which case the ability to distinguish nest-mates from non-nest-mates may be a cost-effective rule-of-thumb to ensure that altruism is directed on average towards kin [2], and aggression towards non-kin [3]. In support of this hypothesis, numerous eusocial hymenopterans [4–6], some termites [7–11] and eusocial mole rats [12] exhibit extreme aggression towards non-nest-mates. Intense and violent intergroup competition is also common in mammalian cooperative breeders [13].

Tests of the role of kin or nest-mate discrimination as a promoter of cooperation in insects have focused mainly on Hymenoptera [6,14,15], while termites, the second-largest eusocial taxon, have been relatively neglected [16–18]. Studies of termites may be particularly illuminating because they vary enormously in colony size and individual specialization [16,19], possess fundamentally different life-history traits from Hymenoptera (such as hemimetabolous development), display radically different feeding ecologies [16], and there are no known extant solitary species [20,21]. Moreover, termites are diploid and hence offer a chance to test the hypothesized role of genetic architecture in the evolutionary origin and maintenance of eusociality [19,20,22,23].

Among extant termites the wood-dwelling species (Termopsidae and Kalotermitidae) are thought to display the most basal life-history traits [24,25] (but see [26]). In contrast to the high morphological and behavioural diversity of derived termites, workers in wood-dwellers tend to show relatively little division of labour and are monomorphic. Most species lack a true worker caste, instead possessing ‘false workers’ or ‘pseudergates’ which are developmentally plastic, and like social vertebrates possess the potential to explore all caste options throughout their lives [17]. In the drywood termites (Kalotermitidae), these ‘workers’ perform little or no brood care or nest maintenance activities [19,27]. However, workers do altruistically feed soldiers (who cannot feed themselves) [18], engage in proctodeal and anal trophallaxis [28] and maintain hygiene [29]. Because the wood these species inhabit serves simultaneously as a source of food, shelter and protection, colony members never voluntarily leave the nest, except as dispersing alates [18,21,30]. However, it has been previously noted in other drywood species that two or more colonies occupying the same piece of wood sometimes meet, which can lead to intergroup aggression and colony fusion [27,30–32].

In this study, we tested the hypothesis that drywood termites can recognize non-nest-mates and direct specific behaviours towards them. We performed a series of behavioural assays using captive colonies of the drywood termite *Pterotermes occidentis* (Kalotermitidae), indigenous to the Sonoran desert of the southwest USA [21]. Specifically, we introduced single individuals into foreign nests and observed and measured subsequent interactions, such as allogrooming rates, which we interpreted as a cooperative behaviour, and frequency of butting, which in other termites is taken as a measure of aggression or dominance [33]. While other aggressive interactions (such as wing pad biting) have been observed in wood-dwelling termites [24], butting was the only potentially aggressive behaviour observed in our study. We predicted that foreign individuals would face increased aggression and receive less allogrooming than individuals native to their own colony, providing evidence of discrimination between nest-mates and non-nest-mates.

## 2. Material and methods

Whole colonies of *P. occidentis* termites were collected from standing *Cercidium floridum* (blue palo verde) trees in September 2011 and October 2013 in the Sonoran desert, Arizona. Of the six colonies used in the experiment, five were collected in one region (Mendoza Canyon: 31.972088° N, 111.470339° W) and one colony was collected within the Tucson metropolitan area (32.273160° N, 110.905818° W). In the Sonoran desert, single colonies of *P. occidentis* stage multiple (up to 40) dispersal flights on nights in July and August [21]. Dispersal distance is unknown for this species, but in other Kalotermitidae alates disperse up to a few kilometres, apparently as an inbreeding avoidance mechanism [34,35]. The five colonies collected from Mendoza Canyon were all located at least 500 m apart when found, and consequently are likely to be founded by unrelated queens. Behavioural observations were conducted between December 2013 and July 2014. During the experiment, all colonies were housed in plastic boxes containing tightly arranged blocks of *C. floridum* wood from the colonies sites of origin and kept in an incubator at a constant temperature of 26 ± 2°C, with 30–36% humidity and permanent darkness.

Fifteen size-matched treatment--control pairs of individuals were selected randomly from each colony and marked with a unique tricolour code using enamel-based paint on the head, thorax and abdomen. Focal individuals were marked 3 days prior to commencement of observations, allowing time for recovery from the procedure. Marking was carried out under anaesthesia following exposure to 30 s of CO_2_ [36]. During the 3-day period between marking and the first observations, all termites were returned to their native colonies in captivity.

**Figure 1.**
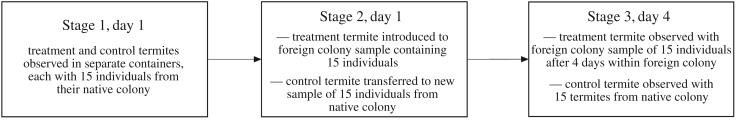
Design and sequence of the experiment.

**Figure 2.**
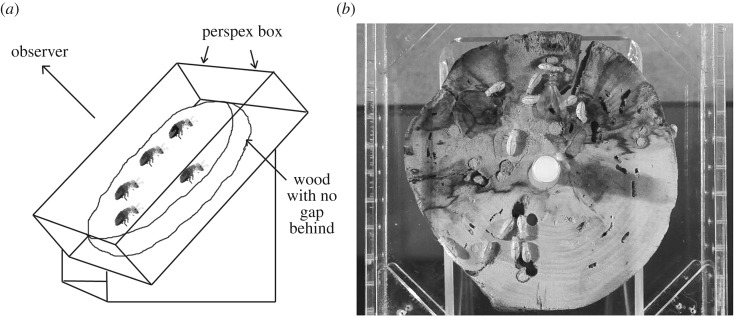
(*a*) Diagram of the experimental set-up as it was during observations. (*b*) Still image of the arena taken from one of the observation videos.

Prior to each observation session, a random subset of 15 termites was selected from either the home or foreign colony with which interactions could take place, depending on the stage of the trial. In total, 40-min behavioural observations were conducted over four stages for each focal termite during a 4-day period (figure 1). Observations were conducted at 27 ± 2°C in Perspex observation arenas separate from the main colony containers, and contained a single, colony-specific piece of *C. floridum* wood to avoid cross-contamination of odour-carrying hydrocarbons (figure 2). The limitation of this set-up is that it differed from the termites' usual tightly confined tunnel networks. However, it did allow for easy observation of individuals, and we observed typical behaviours such as eating and trophallaxis similar to those observed in the main colony housing boxes, suggesting low levels of disturbance in the observation arena. Treatment and control groups were observed simultaneously using video cameras, and observation sessions were preceded by a 30-min calming period following transfer from the nest-box. Focal termites were placed into the observation arenas with colony subsets immediately prior to the onset of this calming period and allowed to settle together.

Allogrooming was measured by timing the onset and end times of each occurrence in seconds and summing the total for each 40-min session. For butting behaviour, which we defined as a vigorous shaking motion in response to disturbance or as a signal of reproductive dominance [33], frequency data were recorded. Occasional periods when the focal termite was hidden or obscured were deducted from each session time. For both behaviours recorded, the focal termite could either be the actor or recipient, and the distinction was noted.

We used general linear mixed-effects models (GLMMs) to predict how treatment affected the proportion of time spent being groomed and frequency of butting interactions. Models were fitted with stage of experiment and treatment as fixed effects, and in our models of butting we also included observation time (which occasionally was less than 40 min due to the focal individual being obscured). In all models, we used individual, native colony of focal termite, and colony sample in which the observation was taking place as random intercepts. In our models of butting behaviour, we also included these observation level random effects to control for overdispersion [37].

The response variables in grooming observations were the proportion of time spent being groomed or performing grooming, and those models were fitted with a binomial error structure. The response variables in butting models were the frequency of received/performed butting observed, using a poisson lognormal error structure. We then conducted post hoc Tukey comparisons between treatment and control groups in each stage to control for multiple pairwise comparisons. To test whether genetic relatedness between colonies (which could conceivably be >0 for Mendoza Canyon colonies) influenced our results we conducted a post hoc Mann–Whitney *U*-test to test whether trials involving focal individuals from the single Tucson area colony differed from those involving focal individuals from Mendoza Canyon colonies.

All analyses were performed using R statistical software [38] using packages lme4 and lsmeans [39,40], with the exception of the Mann–Whitney *U*-tests which were performed in Microsoft Excel 2011.

## 3. Results

**Figure 3.**
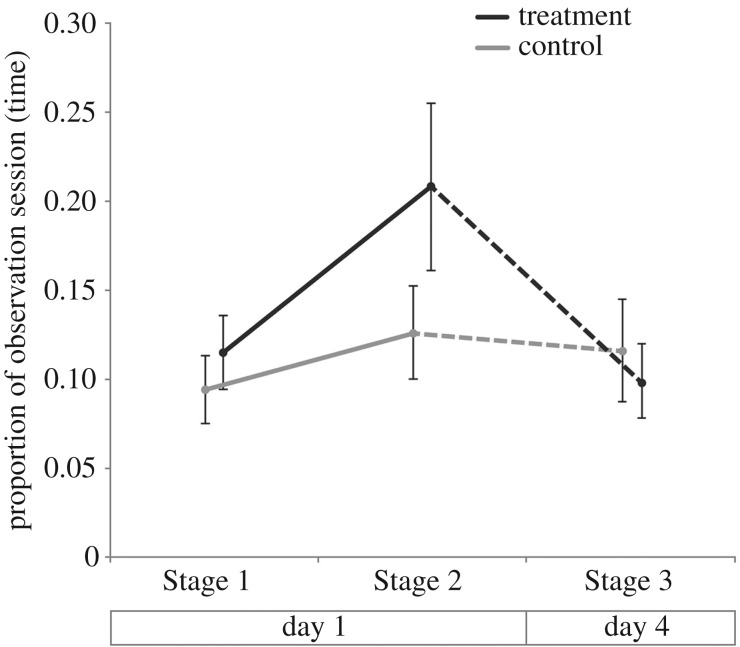
Proportion of observation session for which focal individuals were groomed when introduced to a foreign colony (treatment: black line) or to their own colony (control: grey line). *N* = 15 trials between six colonies in both treatment and control categories. Points show means, bars show standard error.

Treatment affected the amount of grooming the focal individuals received (table 1). Contrary to our predictions, however, grooming towards the foreign individuals increased upon introduction to a foreign colony (Stage 2). This effect disappeared after 4 days within the host colony (Stage 3, figure 3). Post hoc testing revealed no significant difference between trials involving focal individuals from the Tucson area colony and those involving focal individuals from the five Mendoza Canyon colonies (electronic supplementary material). In contrast to our results for grooming behaviour, there was no difference between control and treatment individuals in the amount of butting received or given. Introduction to a foreign colony also had no significant effect on the amount of grooming performed by focal individuals. Finally, there was no significant difference in behaviour (rates of grooming or butting) of treatment or control individuals after reintroduction to their original colony, immediately after Stage 3 (table 1).

**Table 1.** Tukey's honest significant difference tests comparing the allogrooming and butting that control and treatment individuals received and gave in each stage. In each stage, the parameter estimates for treatment individuals are compared with control individuals.

		acts received				acts given			
		*β*	s.e.	*z*	*p*-value	*β*	s.e.	*z*	*p*-value
grooming									
Stage 1	C	−2.53	0.44			−3.52	0.67		
	T	0.03	0.22	0.14	0.44	−0.013	0.82	0.017	0.49
Stage 2	C	−2.24	0.44			−4.07	0.67		
	T	0.75	0.22	3.34*	<0.01*	0.91	0.82	−1.11	0.134
Stage 3	C	−2.31	0.44			−3.59	0.67		
	T	−0.11	0.23	0.51	0.31	−0.5	0.82	0.62	0.27
reintroduction	C	−2.07	0.44			−3.39	0.67		
	T	0.34	0.22	1.49	0.07	−0.64	0.82	0.78	0.22
butting									
Stage 1	C	1.7	0.55			1.27	0.62		
	T	−0.041	0.49	0.084	0.46	−0.7	0.83	0.84	0.2
Stage 2	C	1.57	0.55			0.45	0.66		
	T	0.4	0.49	0.82	0.21	−0.24	0.86	0.28	0.39
Stage 3	C	1.65	0.55			1.29	0.63		
	T	0.28	0.49	0.56	0.29	−0.54	0.83	0.65	0.26
reintroduction	C	1.59	0.54			1.24	0.63		
	T	−0.03	0.48	0.061	0.48	−0.55	0.82	0.66	0.25

*p < 0.05.

## 4. Discussion

The intense levels of grooming directed towards individuals from foreign colonies suggests that *P. occidentis* workers can distinguish nest-mates from non-nest-mates. However, the results were opposite to our original predictions: foreign intruders were not subject to greater levels of aggression (butting), and were the beneficiaries of significantly higher levels of allogrooming, which in social organisms is usually interpreted as a form of cooperation. After 4 days, the rate at which intruders were groomed had dropped back to the same rate as control individuals in their native colony (figure 3).

A plausible explanation for these results is that *P. occidentis* workers use allogrooming to maintain a recognizable colony odour, most probably mediated by cuticular hydrocarbons (CHCs). In fact allogrooming behaviour may be the primary, and perhaps sole mechanism of transferring in-group chemical profiles between colony members [5,18]. Over the course of the 4 days spent integrating with the foreign host colony, elevated levels of allogrooming received by an intruder may reduce any dissimilarity in CHC profile between itself and the members of its host colony, so that by day 4 of our experiment the foreign termite is no longer recognized as an intruder. This hypothesis would predict that reintroduction to the focal individual's own colony should again lead to elevated levels of grooming compared to controls. In our limited sample, levels of grooming upon reintroduction were elevated for treatment individuals, but not significantly so (*p* *=* 0.07; table 1). To test this hypothesis further will require experiments using non-destructive CHC sampling techniques (such as the use of solid phase microextraction (SPME) fibres [41]) to determine whether the profile of excluded individuals drifts from that of their native colony over time; and whether allogrooming functions to homogenize the CHC profile of intruders.

Why should *P. occidentis* group members actively try to integrate foreign individuals into a colony (through allogrooming), rather than repelling or attacking them as commonly occurs in many insect and vertebrate societies? Encounters between colonies may be frequent in wood-dwelling termites such as *P. occidentis,* because suitable nesting trees usually contain multiple colonies [30]. With colony growth and expansion of nest galleries, colony contact can occur when adjacent cavities meet [22,23,27,32]. Genetic studies of within-colony relatedness in wild populations have found evidence for mergers in several species of both wood-dwelling [27,32,42–44] and external foraging termites [45–49], with one study finding evidence of multiple mergers in several colonies of the drywood termite, *Kalotermes flavicollis* [32]. In laboratory studies of the drywood termite, *Cryptotermes secundus*, peaceful colony mergers are associated with increased colony survival and an increased production of new reproductives, suggesting that individual workers may stand to benefit (in terms of direct fitness) when colonies fuse [27]. Similarly, in the dampwood termite, *Zootermopsis nevadensis*, colony fusion creates opportunities for workers to inherit reproductive status [30]. Theoretical models predict that an increase in the probability of inheritance should favour lower investment in self-sacrificial, colony-beneficial behaviour, such as intergroup conflict [50,51] (note this prediction does not necessarily hold if invaders represent a threat to an individual's inheritance rank [52]). In addition, as the indirect fitness benefits of helping appear to be relatively low in wood-dwelling termites [18], workers have less incentive to invest in colony defence to maintain kinship within groups at the expense of their own potential future fecundity. Together, these ecological and social factors may explain why in wood-dwelling termites individual workers may be selected to detect and integrate foreign individuals into the group rather than attack them.

An alternative hypothesis to explain our results is that foreign individuals are perceived as potentially harbouring pathogens, with allogrooming being an adaptive, selfish response to maintain social immunity and colony health [53]. Rosengaus *et al*. [29] observed that drywood termites tend to have low pathogen loads, but whether this is linked to allogrooming behaviour is unknown. In dampwood termites, elevated levels of allogrooming behaviour have been observed following exposure to fungal pathogens [29,54], and substantially lowered external pathogen loads have been observed following experimental exposure to fungal spores and subsequent grooming [46]. Experimental manipulation of pathogen load in *P. occidentis* could be used to test this hypothesis.

In summary, the basal life-history traits of *P. occidentis* [31] make the species an excellent system to investigate the behavioural factors and evolutionary processes associated with the apparently unique origin of termite sociality. Future fitness benefits, low levels of helping behaviour, and strict ecological and temporal constraints on colony growth may explain why relationships between groups in basal termites are less fractious and violent than is typically the case in eusocial Hymenoptera and cooperatively breeding vertebrates. We suggest that further studies of intergroup interactions in basal termites may contribute to an improved understanding of the role of between-group competition in social evolution.

## Supplementary Material

Supplementary Information

## Data accessibility

Our data are deposited at Dryad: http://dx.doi.org/10.5061/dryad.tb0c7 [55].
