# Supplementary material for: Lack of aggression and apparent altruism towards intruders in a primitive termite
Source: R Soc Open Sci. 2016 Nov 9;3(11):160682. doi: 10.1098/rsos.160682 (PMC5180156; doi:10.1098/rsos.160682)
Supplement: Supplementary Information [file rsos160682supp1.docx]

Supplementary Information:

Our experiment used individuals from 5 colonies at one site (Mendoza Canyon), together with 2 trials using focal individuals from a single colony (colony 1) in the Tucson area, over 100km away. As such it is possible that relatedness between focal individuals and the foreign colony was much lower in the trials involving colony 1. To investigate this possibility we performed a Mann-Whitney test to determine whether data from the 2 colony 1 trials differed significantly from the other trials involving only Mendoza Canyon colonies. The Tucson trials did not differ from Mendoza trials at any stage of the experiment: Stage 1: U=2, p=0.98, Stage 2: U=13, p=0.53, Stage 3: U=12, p=0.46. For the Mendoza canyon trials n=13. No valid z-values can be given due to the small sample size in the Tucson area trials (n=2).

Supplementary Figure 1. Received allogrooming in trials for which the focal individual was from the single colony extracted from the Tucson area (‘colony 1’) versus trials where the focal individual and foreign colony were both extracted from Mendoza Canyon area (‘all other colonies’). The Y-axis represents the proportion of observation session for which focal individuals were groomed when introduced to a foreign colony. Lines connect means, vertical bars show standard error.
